# Heart Failure and Main Pulmonary Artery Obstruction Caused by Right Ventricular Metastatic Adult Hepatoblastoma: A Case Report

**DOI:** 10.1097/MD.0000000000001535

**Published:** 2015-09-18

**Authors:** Gongcheng Huang, Liliang Shu, Jing Xu

**Affiliations:** From the Department of Cardiovascular Surgery, The First Affiliated Hospital of Zhengzhou University, ZhengZhou, Henan, P. R. China.

## Abstract

We report a case of a 18-year-old boy who presented with dyspnea and right upper quadrant abdominal dull pain. According to urgent echocardiography, a dense sessile mass occupied the right ventricule. Tumor resection was performed, followed by further adjuvant therapy. The specimen was histopathologically investigated and eventually diagnosed as metastatic adult hepatoblastoma. We discuss its clinical features and treatment in the light of the current knowledge.

It is important for us to be aware that adjuvant chemotherapy might be an effective alternative in the treatment of hepatoblastoma combined with ventricle invasion. Early cardiac surgery may be advised in patients with cardiac function impairment.

## INTRODUCTION

Adult hepatoblastoma is a very rare and aggressive neoplasm. According to published medical literatures, only a few cases have been adequately reported so far. For adult hepatoblastoma, the invasion of the right ventricular causing main pulmonary artery obstruction and heart failure is extremely rare. This situation places the patient in a high-risk group and makes treatment strategy more complex. We reported an 18-year-old male patient with a metastatic adult hepatoblastoma invasing right ventricle, and the tumor was successfully removed with cardiac surgery.

## CASE REPORT

**FIGURE 1:**
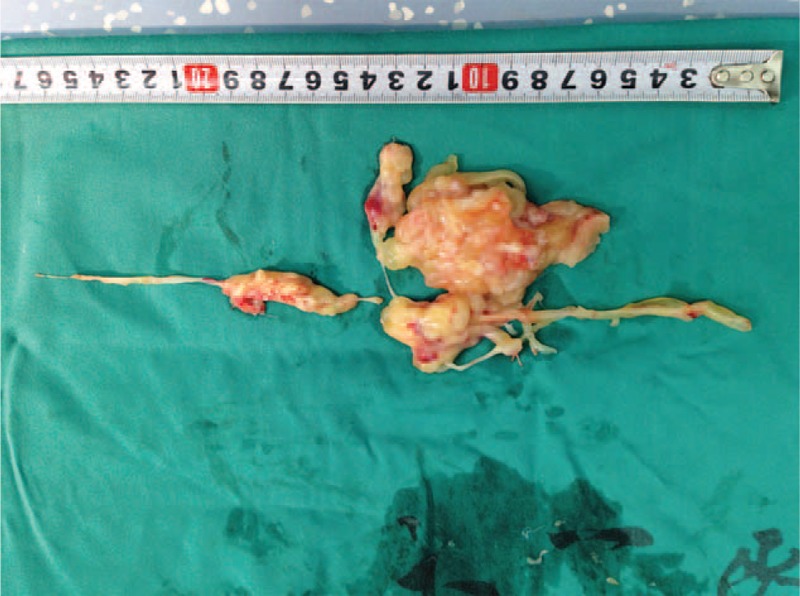
The right ventricule incised and the exposed tumor.

**FIGURE 2:**
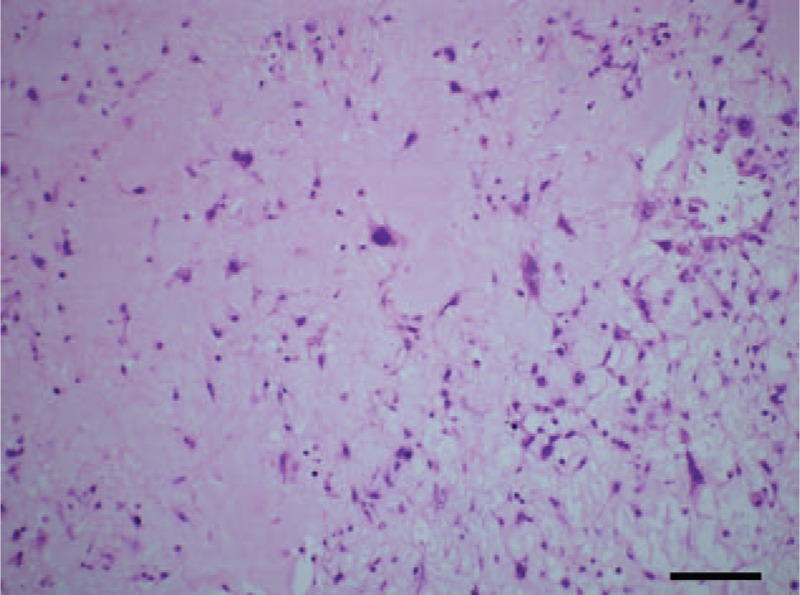
The specimen was histopathologically investigated and eventually diagnosed as rhabdomyosarcoma.

**FIGURE 3:**
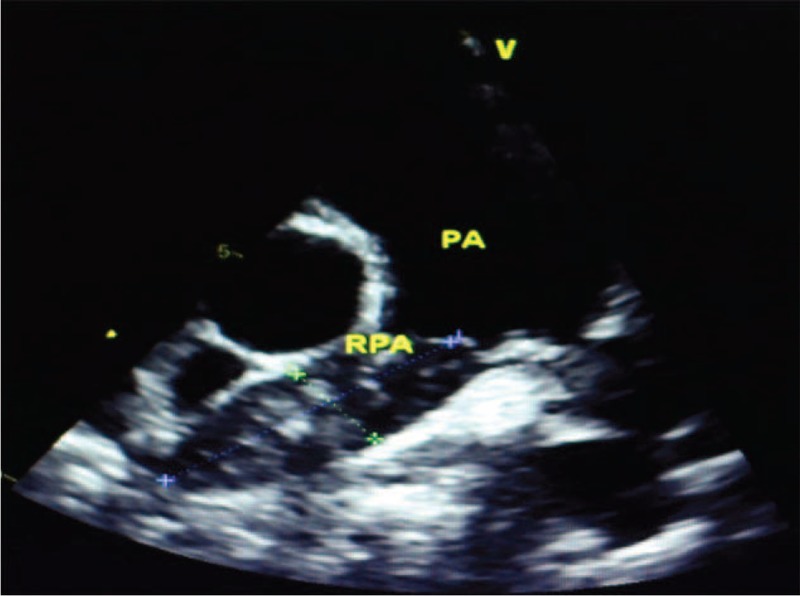
Echocardiography revealed the right ventricule was occupied by a dense sessile mass.

An 18-year-old male patient was referred to the Cardiology Emergency Department with dyspnea and progressive weakness accompanied by cough and edema of lower extremity. One month ago, he had been submitted to hepatectomy due to the primary adult hepatoblastoma at a local hospital. Clinical examination revealed distended jugular veins, systolic murmurs could be heard on tricuspid valve area, edema of lower extremity, and the sign of left side pleural effusion was positive. The serum levels of carcinoembryonic antigen (CEA) were elevated at 239.1U/mL, alpha fetoprotein (AFP) was elevated at 2 ng/mL. Arterial blood gas analysis showed PaO_2_ (arterial partial pressure of oxygen) was 61 mm Hg and PaCO_2_ (arterial partial pressure of carbon dioxide) was 38 mm Hg. Chest X-ray showed diffuse bilateral alveolar infiltrates and left-sided pleural effusion. Urgent echocardiography (Figure 3) revealed the right ventricule was occupied by a dense sessile mass (43 mm × 30 mm) causing right ventricule and main pulmonary artery filling restriction, pulmonary artery hypertension (83 mm Hg), and tricuspid moderate regurgitation (Figure 1). On August 30, 2013, the patient underwent surgical treatment due to worsening of his clinical condition. The large tumor was carefully and copiously dissected from the surrounding tissues due to its friability. The tumor originated mostly from the right ventricle and its terminal end was inside the main pulmonary artery. It was cautiously dissected from the tricuspid valve and the right ventricular endocardium ensuring that no remnants were left behind both on the tricuspid valvular cusps and within the vicinity of the right ventricle. Macroscopically, the tumor appeared sallow and the size was about 10 cm × 8 cm × 2 cm. The specimen was histopathologically investigated and eventually diagnosed as metastatic adult hepatoblastoma (Figure 2). Immunohistochemistry findings showed AFP(−), CK7(−), CK8/18(+), Hepa(−), Vim(+), Syn(−), CD34(+), CD10(−), Ki67(+,80%), S-100(−), NF(−).

After operation, respiratory symptoms of the patient improved significantly, arterial blood gas analysis showed PaO_2_ was 89 mm Hg and PaCO_2_ was 41 mm Hg. Echocardiography revealed pulmonary artery pressure dropped to normal. Then, the boy was referred to the Oncology Department for further adjuvant therapy. Twelve months of follow-up, echocardiography and CT showed there were no signs of metastasis and recurrence. The patient provided written informed consent for the publication of these case details, and the consent procedure was approved by the Human Ethics and Research Ethics committees of The First Affiliated Hospital of Zhengzhou University, Henan, China.

## DISCUSSION

Hepatoblastoma is the most common malignant liver tumor between the age of 6 months and 3 years, but it is extremely rare in adults. Most of these tumors derive from the embryo during liver development and have embryonic features such as numerous mitoses.^1^ There are few reports about giant metastatic adult hepatoblastoma within the right ventricle described in the current literature, especially leading to right heart failure and main pulmonary artery obstruction. Two-dimensional echocardiography is the most common method used to detect cardiac tumors and their complications because of its low cost and high sensitivity.^2^ But the final diagnosis depends on histopathology and immunohistochemistry during surgery.

Complete surgical resection is the primary treatment for patients with hepatoblastoma and is also the only chance for optimal clinical outcome. Furthermore, hepatoblastoma is sensitive to chemotherapeutic agents, such as doxorubicin, cisplatin, vincristine, 5-FU, and cyclophosphamide.^3^ So it is reasonable to select radical resection and chemotherapy for hepatoblastoma in adults.^3^

The prognosis of hepatoblastoma is extremely poor. In adults, the survival durations of reported cases are varied from 2 weeks to 151 months (median survival duration is 6 months). Younger patients had significantly better prognoses than older ones. Patients who underwent resection displayed improved survival compared with those who underwent nonsurgical treatment.^4,5^

This report described a patient with a primary adult hepatoblastoma combined with main pulmonary artery obstruction and heart failure caused by metastasis to the right ventricle. After the urgent surgical treatment with tumor resection, he was referred to the Oncology Department for further adjuvant therapy. Twelve months later, the patient is still alive in excellent condition. It shows that right heart involvement from a malignant tumor must be cautious and should be considered in patients with a history of hepatoblastoma presented with symptoms of right heart failure. It is important for us to be aware that adjuvant chemotherapy might be an effective alternative in the treatment of hepatoblastoma combined with ventricle invasion. Early cardiac surgery may be advised in patients with cardiac function impairment.^6^
